# Towards a new PhD Curriculum for Digital Finance

**DOI:** 10.12688/openreseurope.16513.1

**Published:** 2024-01-08

**Authors:** Lennart John Baals, Jörg R. Osterrieder, Branka Hadji-Misheva, Yiting Liu

**Affiliations:** 1 Institute of Applied Data Science and Finance, Bern University of Applied Science, Bern, Bern, 3005, Switzerland; 2 High-Tech Business and Entrepreneurship, University of Twente, Enschede, Netherlands Antilles

**Keywords:** Digital Finance; Artificial Intelligence (AI); Blockchain Technology; Sustainable Finance; Financial Education and Training

## Abstract

The rapidly evolving field of Digital Finance necessitates a new, interdisciplinary approach to doctoral training. This manuscript presents a comprehensive curriculum designed to equip early-stage researchers with the skills and knowledge required to navigate the complexities of modern finance. The curriculum is structured around four pillars: Training through Research and Mandatory Scientific Training, Advanced Scientific Training, Transferable Skills Training, and Training through Secondments. Together, these pillars provide a balanced mix of theoretical knowledge, practical experience, and soft skills. The program also emphasizes international collaboration through conferences and offers online courses for accessibility and sustainability. By addressing key challenges such as data quality, deployment of complex models, trust in AI-supported products, and labor shortages, the program aims to foster innovation and competitiveness in the European Finance industry. The curriculum’s alignment with the European Digital Finance Package and integration with leading institutions ensures its relevance and potential for significant impact.

## 1 Disclaimer

The views expressed in this article are those of the authors. Publication in Open Research Europe does not imply endorsement of the European Commission.

## 2 Executive summary

The manuscript outlines a comprehensive and innovative doctoral training program in Digital Finance, addressing the multifaceted needs of the European Finance industry. Pillar 1, Training through Research and Mandatory Scientific Training, emphasizes the development of scientific knowledge and skills through original research and foundational courses, serving as the bedrock for the entire program. Pillar 2, Advanced Scientific Training, offers a tailored selection of elective courses, focusing on the intersection of Finance, data science, AI, ML, explainability, blockchain, and sustainability, fostering specialization and innovation. Pillar 3, Transferable Skills Training, ensures the development of essential soft skills, aligning with the 2018 Eurodoc report for early-stage researchers, recognizing the importance of communication, collaboration, and other transferable skills in modern research. Pillar 4, Training through Secondments, provides hands-on experience in world-leading research centers and industry, enhancing understanding of Digital Finance implementation and preparing researchers for practical challenges. In addition to these pillars, the program integrates International Conferences and Other Training Programs, fostering community building and collaboration with external stakeholders. Online courses are also a vital component, ensuring accessibility and long-term sustainability of the training materials. Together, these elements form a robust and forward-thinking curriculum, preparing researchers to tackle the complex challenges of Digital Finance and contribute to the global competitiveness of the European Finance industry.

## 3 Introduction

The rapidly evolving field of Digital Finance is transforming the financial landscape, introducing new opportunities and challenges that extend beyond traditional financial education. With the emergence of Artificial Intelligence (AI), Machine Learning (ML), Explainability of AI (XAI), Blockchain applications, and sustainable finance, there is a growing need for specialized training and research that can bridge the gap between academia, industry, and policy-making.

The European Finance industry, in particular, faces several key hurdles as it strives to compete on a global scale. These challenges include data quality issues related to the increasing complexity of financial data, deployment issues of complex models in real-world applications, deficits in trust and user adoption of AI-supported financial products, potential biases in AI models, and a shortage of talent with the necessary skills to drive digitization efforts in finance ( WEF, 2020) ^1^.

DIGITAL, an Industrial Doctoral Network, is designed to address these challenges by training young researchers in the multidisciplinary aspects of Digital Finance. The network aims to foster innovation and develop solutions to the specific hurdles identified by the industry, including data quality, deployment of AI and ML models, trustworthiness of AI, Blockchain applications, and sustainable finance. The research program within DIGITAL aligns with the strategic priorities of the European Union, focusing on areas such as data quality, deployability of AI models, explainability and trustworthiness of AI, Blockchain technology, and sustainability [( European Commission (EC), 2023) ^2^; ( European Green Deal, 2019) ^3^; ( The European Commission’s Blockchain strategy, 2021) ^4^]. The program’s structure, encompassing five interconnected work packages (WPs) and 17 Individual Research Projects (IRPs), ensures a comprehensive and integrated approach to Digital Finance.

Furthermore, DIGITAL’s commitment to creating a new qualification standard and a long-term sustainable PhD program aligns with the European Digital Finance Package and the MSCA guidelines on supervision [( EC, 2020) ^5^; ( EC, 2021) ^6^]. This alignment underscores the network’s role in enhancing the competitiveness of the European industry in the strategic domain of Digital Finance and accelerating the digital and green transition of Europe. By providing specialized training and fostering collaboration across sectors, DIGITAL aims to equip researchers with the skills and insights needed to drive innovation and contribute to the future of Digital Finance.

## 4 Soundness of the proposed methodology

### 4.1 Overall methodology

The planned doctoral network follows an approach of broad comprehensive training complemented by cutting-edge research projects executed by 17 ESRs. Researchers will not only develop key scientific skills and propose novel technologies that are on the frontiers of big data-, AI-, blockchain- and sustainability research, but they will also build easily deployable solutions to the key hurdles that finance service providers face in using these technologies in production, disseminating this to all stakeholders, incorporating feedback from industry and policy makers.

The central research question in DIGITAL is how innovative technologies, like big data, artificial intelligence and blockchain, can be used to support Digital Finance in view of the emerging complexities: (i) high-dimensional, high-variety, high-velocity dataset; (ii) limited samples of high-quality data to train various ML models, (iii) no comprehensive pipeline for building and deploying complex ML models in real settings, (iv) no explainability techniques that are specifically tailored to financial datasets and satisfy the explainability needs of various financial stakeholders, (v) no industry standard for blockchain applications and (vi) no common ESG scoring framework.

All of these complexities are methodological in nature and in order to tackle them, DIGITAL will carry out pragmatic, data-focused, inductive research using a combination of research strategies (case studies, experiments and actions) and methods enriched with continuous cooperation with and feedback loop from industry and regulators. We elaborate on the central layer of the research methodology, which combines academia, industry, dissemination, and training with seven building blocks.

1. **Data.** All research objectives heavily depend on the networks’ access to financial data (both structured and unstructured) for the different functions of finance: trading (including new assets like cryptos), personal lending, Small- and Medium-sized Enterprise (SME) lending and sustainable investing. Data has already been made available primarily by our large consortium of partner universities and industry partners, which possesses a plethora of datasets that have not yet been fully exploited for academic research and business use. Additionally, researchers will be encouraged to carry out primary data collection as well i.e. collect and generate new relevant datasets (eg. through web scraping, surveys and questionnaires).
2. **Preliminary research & specificity.** The network, independently from this proposal, has already collaborated extensively through different cooperation. As a result, a significant amount of preliminary work has been done within the research objectives which has further defined (to a certain extent) the envisioned research methodology for the IRPs.
3. **Two-phased methodological approach.** DIGITAL will emphasise a mixed-method approach, resulting in comprehensive research findings that incorporate qualitative and quantitative data signals. Typically, each IRP consists of two stages. In the initial phase, we apply existing methods to new datasets and transform them into prototypes and use cases so that new financial products can be developed. Once we have these Minimum Valuable Products (MVPs), we will conduct additional research to achieve new breakthroughs and methods in a second phase. Nonetheless, the precise selection will depend on the IRPs’ underlying objectives:
  • WP 1. Towards a European financial data space. In order to address the growing complexity of financial data, researchers will use natural language processing (NLP), transformers and attention networks to incorporate text and identify temporal dependencies in various financial use cases. On the topic of data augmentation, recruited researchers will look at convolutional networks with attention and transformers for simulating various financial time series.
  • WP 2. AI for financial markets. In order to address some of the key deployment issues of AI models in real financial use cases, researchers will work on: simulating the real market environment in deep reinforcement learning applications; produce dynamic scoring systems based on complex ML models (XGBoost, random forest, SVM, neural nets etc.) that are able to deal with sparse credit history of loan applicants; and apply (unrestricted-) mixed data sampling ((u-) midas) methods so to align different frequency financial data.
  • WP 3. Towards explainable and fair AI-generated decisions. In order to validate the utility of classical XAI methods for finance, recruited researchers will map the state-of-art post-hoc global and local explainability techniques (LIME, SHAP, LRP) to the explainability needs of finance stakeholders. IRPs will also focus on developing new XAI methods for time series that mark preference for the sensitivities or partial derivatives for each explanatory variable included in the ML’s specification, thus excluding the need for perturbation. Finally, to tackle the challenges surrounding algorithmic bias, disparate learning processes (DLPs) and the variational fair Autoencoder, will be applied.
  • WP 4. Driving digital innovation with Blockchain applications. Within WP4, DIGITAL will propose a novel methodology for industry-level blockchain standard which includes comparison criteria, documentation criteria and content criteria. Furthermore, in order to tackle the emerging risks from Blockchain applications in finance (volatility of crypto assets and fraud), researchers will develop a dynamic crypto risk index based on a model selection criteria, and propose network-community detection models so to explore the transactional crypto network and uncover ambiguous links.
  • WP 5. Sustainability of digital finance. Within DIGITAL, recruited researchers will explore, train and compare content-based, collaborative and hybrid filtering approaches in the attempt to find the most suited recommender system to support financial institutions and customers on investing in sustainable technologies. Yet another methodological challenge tackled within WP 5 is that of building unifying and comprehensive green credit scores for retail and business clients.
4. **Secondments, continuous cooperation and feedback loop.** Continuous collaboration and feedback loops with industry and other relevant non-academic partners are a defining characteristic of the DIGITAL research model (ECB, ARC and FRA). Each IRP is established jointly by academic and industry representatives, and each ESR will spend 18 months in industry and 4 months in world-class research centres and government agencies. With this design, we ensure that the progress with respect to the established research objectives is continuously monitored, validated by industry, and in accordance with the existing and upcoming technological regulations. The continuous feedback loop with industry ensures that developed solutions are applicable in the real world.
5. **Dissemination and communication methods.** Our methodology relies on substantial dissemination and communication efforts (such as more than 120 academic events organised by our extended network since 2020), which will enable us to collect feedback, stay abreast of the most recent developments, and shape the future of this research direction. ESRs receive extensive feedback from academics and other stakeholders, allowing them to expand or shift their research focus and methods, as well as validate the effectiveness of their approaches.
6. **Integration into doctoral training.** Our methods also anticipate feeding back into doctoral education. All ESRs will routinely present their findings at DIGITAL seminars, and supervisors will be expected to incorporate new findings into doctoral training programs. This will be accomplished primarily through the new modules developed by the network which will be continuously updated to include the new methods, technologies, solutions, tools, and business models created by the network.
7. **Cooperation with leading institutions.** Our methodology is also strongly based on working together with very large networks of researchers (more than 400 academics in COST, 170 institutions in ECMI) to collect feedback and learn the latest techniques, as well as leading institutions, such as the European Central Bank.

**Challenges.** There are both data availability and methodological challenges. Data has already been used, but if some new data cannot be shared easily, we will provide specialised legal solutions (NDAs), and acquire the required licences. Additionally, in case the problem remains unsolved, we will employ data anonymization techniques that enable data sharing or resort to synthetic data generation techniques (GANs). On the methodology-related challenges, in cases in which the planned techniques do not lead to the envisioned outcomes, we will expand the scope of models to be trained and tested and reevaluate the planned effort. In case of any deployment issues (i.e. the developed methodologies are not fully suited to the IT constraints in which finance service providers operate), we will offer the solution as a standalone product rather than as an incorporated service.

## 5 Overview and content structure of the doctoral training programme

DIGITAL will produce a substantial amount of new doctoral training materials that will be taught at network-wide events, made available to all (for detailed course descriptions please refer to Table 3 and Table 4).

### 5.1 Pillar 1: Training through research and mandatory scientific training

Each recruited researcher within the DIGITAL network will embark on a comprehensive journey to develop new scientific knowledge and skills through conducting original research under their Individual Research Projects (IRPs). This training pillar is designed to foster innovation, creativity, and expertise in the fields of big data, artificial intelligence, blockchain, and sustainability research.

***5.1.1 Mandatory foundation courses.*** The training begins with five mandatory foundation courses (local training, Table 1.3.a). These courses are carefully curated to provide the essential theoretical background and practical skills required for the researchers’ specific areas of study. The courses may include topics such as:

- **Introduction to digital finance:** An overview of the key concepts, technologies, and trends in digital finance.
- **Machine learning and artificial intelligence:** A deep dive into algorithms, models, and applications of machine learning in the financial sector.
- **Blockchain technologies:** Exploration of the principles, applications, and challenges of blockchain in digital finance.
- **Sustainable finance and ESG principles:** Understanding the role of sustainability and ESG (Environmental, Social, Governance) principles in modern finance.
- **Research methodology and ethics:** Guidance on conducting ethical and methodologically sound research in the field of digital finance.

***5.1.2 Network training events.*** In addition to the local training, ESRs will participate in mandatory network training events ( Table 1.3.b). These events are designed to foster collaboration, knowledge sharing, and professional development across the network. Key features of the network training events include:

- **Collaborative workshops:** Opportunities for researchers to work together on shared challenges, exchange ideas, and learn from each other’s expertise.
- **Guest lectures and seminars:** Presentations from leading academics, industry experts, and policymakers to provide insights into cutting-edge research and real-world applications.
- **ESR presentations:** A platform for ESRs to present their research findings, receive feedback, and engage in constructive dialogue with peers and mentors.
- **Skill development sessions:** Tailored sessions to enhance soft skills such as communication, teamwork, and project management, essential for a successful research career.

**Table 1.** Table 1.3.a Main local training courses.

#	Course	WP	Description	ECTS	Month
1	Foundation of data science (BBU)	1	Introduction to a range of topics and concepts related to the data science process. It will cover the technical pipeline from data collection, to processing, analysis and visualisation.	4	M12
2	Introduction to AI for financial applications (WWU)	2	Getting started with ML; Introduction to supervised and unsupervised learning; deep learning and reinforcement learning. Explore how to use these methods for financial applications (financial forecasting, credit risk, etc.)	4	M12
3	The need for eXplainable AI: methods and applications in finance (BFH)	3	Introduction to XAI methods; state-of-art models (LIME, SHAP, DiCE, LRP, counterfactual explanations, etc.). Challenges of classical methods. Introduction to methods suited for financial applications.	4	M12
4	Introduction to Blockchain applications in finance (HUB)	4	Introduction to the blockchain technology, concepts such as mining, hashing, proof-of-work, public key cryptography, and the double-spend problem. Overview of the design principles and challenges.	4	M18
5	Sustainable finance (UNA)	5	Introduction to sustainable finance strategies. Overview of how these strategies can minimise organisational risk, create long-term business value, and improve social and environmental impact.	4	M18

*Notes*: This table outlines the main local training courses offered in the DIGITAL program. It includes details on the course name, associated work package (WP), a brief description of the course content, the European Credit Transfer and Accumulation System (ECTS) credits awarded, and the month in which the course is scheduled (M12 refers to month 12, etc.).

**Table 2.** Table 1.3.b Main network-wide training events, Conferences and Contribution of Partners.

#	Main Training Events & Conferences	ECTS	Lead	Action Month
1	Kickoff Meeting and Technical Training – University of Twente (NL) [fellows, academic- and industrial supervisors, and representatives from associated partners] Visits to the Finance Lab of the university will be arranged, where researchers will be offered several training sessions like “Introduction to Digital Finance”, “Blockchains in Digital Finance”, and “Ethical AI in Finance” by the UT faculty and additional consortium members.	3	UTW	M12
2	Orientation Training Digital Finance - WU Vienna (AT) [doctoral training combined with COST Action meeting] This meeting will be organised in conjunction with a COST Action meeting, enabling fellows to meet European researchers in Digital Finance outside the doctoral network. The meeting will be preceded by a general five-day training course in Digital Finance.	3	WWU	M15
3	Industrial Doctoral School on FinTech - EIT Digital (ES) [open summer school hosted by EIT Digital] EIT Digital will organise an open summer school in Madrid, tailored to doctoral candidates in FinTech. Fellows will be able to interact with doctoral candidates in digital finance from outside the network. The theme of the summer school is “Disrupting Finance with Digital Technologies”.	4	EIT	M20
4	Regulation in Digital Finance Workshop – European Central Bank (EU) [doctoral training combined with ECB site visit] The workshop includes site visits to the ECB, providing direct immersion into the regulatory aspects of digital finance, including topics such as AI bias, data sovereignty and digital currencies. Fellows will attend a four-day training program. Two days are allotted to the topic of ‘Regulatory aspects of digital finance’, conducted by regulatory experts from ECB.	2	ECB	M24
5	Mid-term Review Event - Babes,-Bolyai University (RO) [fellows, academic- and industrial supervisors, and representatives from associated partners] The mid-term review event is a key event for the DIGITAL network that will bring together researchers, industry, regulators and supervisors. The training component of the meeting is a short course on “Sustainability in Digital Finance”.	1	BBU	M30
6	Digital Finance Industry Event - University of Naples (IT) [hosting industrial partners from within and outside the doctoral network] Fellows will intensively interact with industry and orient themselves on potential digital finance careers outside academia. Industrial partners from both within the network (DEL, CAR, INT) and outside will provide training on: “Fraud detection in digital accounting”, “Responsible AI in finance” and “Sustainable digital finance”.	2	UNA	M36
7	Training & Development Workshop - Kaunas University of Technology (LT) [fellows, academic- and industrial supervisors, and representatives from associated partners] In addition to an open conference on the topic of ML for Option Pricing, the event will also include content training on “Designing digital finance tools” and a transferable skills training on “Entrepreneurship in digital finance”.	2	KUT	M40
8	Closing Conference – University of Twente (NL) [fellows, academic- and industrial supervisors, and representatives from associated partners] A selection of renowned keynote speakers from both academia and industry will speak at plenary sessions. Fellows will also have the chance to speak with principal scientists and industrial partners, reflecting on their work. The best project of the doctoral network, selected by the advisory board, will receive a Best Doctoral Research award.	2	UTW	M48

*Notes*: This table summarizes the main network-wide training events, conferences, and contributions of partners in the DIGITAL program. It includes details on the event name, European Credit Transfer and Accumulation System (ECTS) credits awarded, the lead organization, and the scheduled action month. The events encompass various aspects of digital finance, including technical training, orientation, regulation, industry interaction, and closing conferences.

**Table 3.** Courses (E existing, N - new module).

#	Courses (E existing, N - new module)	WP	Tutorial content	Lead	ECTS	Month
1	Synthetic Data Generation for Finance (N)	1	Use of deep learning techniques (e.g., Generative Adversarial Networks) to generate synthetic financial data indistinguishable from real data. Use cases for synthetic data in AI training, e.g., fraud detection, crisis simulation.	ARC	4	M12
2	Anomaly Detection in Big Data (E, N)	1	Principles to detect anomalies. Discuss ways of handling data errors (e.g., human inspection, removing outliers, deploying AI to fill in gaps in data). Mapping of data quality.	BBU	4	M18
3	Natural Language Processing with Transformers (E, N)	1	Combine computational linguistics and role-based modelling of human language with statistical machine learning and deep learning models. Understand to use the most advanced transformers to perform advanced tasks.	ARC	4	M24
4	Dependence Structures in High Frequency Financial Data (N)	1	Automatic detection of dependencies between arbitrary numbers of vectors. Techniques for identifying patterns such as time-dependent trends, volatility clustering, seasonality, and fat tails. Application of copulas and spectral measures.	ASE	3	M30
5	Reinforcement Learning in Digital Finance (N)	2	Selection of learning algorithms relevant to digital finance applications. Deploying RL for decision-making in areas such as trading, risk management, and fraud detection.	UTW	4	M12
6	Machine Learning in Industry (N)	2	Principles of machine learning in industry. Business assessment of automation decisions. Practical implications of machine learning. Availability and costs of high-quality data.	CAR	4	M18
7	Deep Learning for Finance (E, N)	2	Build and train deep neural networks, identify key architecture parameters, implement vectorized neural networks and deep learning. Analyse variance for DL applications.	BBU	3	M24
8	Data-Centric AI (N)	2	Empower SMEs in digital finance to deploy AI with limited datasets. Construct high-quality samples to maximise training impact. Identify weak spots in data quality.	WWU	3	M30
9	Cybersecurity in Digital Finance (N)	3	Cybersecurity from perspectives of social behaviour, software and hardware. Security of cloud services and compliance with EU regulations. Detecting and preventing fraud.	UTW	3	M12
10	AI Design in Digital Finance (N)	3	Overview of contemporary AI techniques in digital finance. Designing impactful AI with explicit consideration for energy consumption, bias, explainability, and fairness.	HUB	4	M18
11	Barriers in Digital Finance Adoption (N)	3	Hurdles for society-wide adoption of digital finance. Design principles to include genders, minorities and vulnerable groups. Fast-paced industry, start-up climate, competition.	WWU	3	M24
12	Explainable AI in Finance (E, N)	3	Classification of white box- and black box models. Applicability of classical XAI techniques in finance. Audience-dependent explanations. Emerging XAI techniques.	BFH	4	M30

*Notes*: This table outlines the courses offered in the program, including both existing (E) and new (N) modules. It details the course name, work package (WP), tutorial content, lead organization, European Credit Transfer and Accumulation System (ECTS) credits awarded, and the scheduled month. The courses cover a wide range of topics in digital finance, including synthetic data generation, anomaly detection, natural language processing, deep learning, cybersecurity, and regulation.

All network training events will feature presentations from ESRs, allowing them to showcase their progress, articulate their ideas, and contribute to the collective knowledge of the network. This collaborative and multifaceted approach ensures a well-rounded training experience that prepares researchers for excellence in both academia and industry.

### 5.2 Pillar 2: Advanced scientific training

Pillar 2 focuses on providing each Early Stage Researcher (ESR) with advanced scientific training tailored to their individual experience, research projects (IRPs), and career aspirations. This pillar is designed to deepen the researchers’ expertise and prepare them for cutting-edge research and innovation in the rapidly evolving fields of Finance, data science, AI, machine learning (ML), explainability, blockchain, and sustainability.

***5.2.1 Elective advanced courses.*** Each ESR will be assigned a combination of at least three elective advanced courses. These courses are made accessible to a wider audience and are carefully selected to align with the fellow’s specific research focus and career goals. Examples of elective advanced courses may include:

- **Advanced machine learning in finance:** Exploration of complex ML algorithms and their applications in predictive modeling, risk management, and financial analysis.
- **Blockchain and cryptocurrency technologies:** In-depth study of blockchain protocols, consensus mechanisms, and the development of decentralized financial applications.
- **Sustainable finance and impact investing:** Examination of sustainable investment strategies, ESG integration, and the role of finance in achieving global sustainability goals.
- **Explainable AI and ethical considerations:** Understanding the principles of explainable AI and the ethical implications of AI-driven decision-making in the financial sector.

***5.2.2 Creation of new modules.*** Recognizing the dynamic nature of the field, many new modules will be created at the intersection of Finance, data science, AI, ML, explainability, blockchain, and sustainability. These modules will address emerging challenges and opportunities that are not covered in existing Finance PhD programs. The development of these new modules ensures that the training remains at the forefront of technological advancements and industry trends.

***5.2.3 Collaboration with cooperation partners.*** In addition to the elective courses and new modules, the training program will leverage collaborations with cooperation partners such as the COST Action CA19130 ( COST CA19130, 2023) ^7^, EIT Digital, and ECMI ( ECMI, 2023) ^8^. These partnerships will provide access to specialized doctoral courses, summer schools, workshops, and conferences. By opening up these opportunities to all recruited ESRs, the program fosters a rich learning environment that extends beyond the traditional academic setting.

***5.2.4 Tailored training experience.*** The combination of elective advanced courses, innovative new modules, and collaboration with renowned partners creates a tailored training experience for each ESR. This personalized approach ensures that researchers are equipped with the knowledge, skills, and insights needed to excel in their research endeavors and contribute to the advancement of digital finance and related fields.

### 5.3 Pillar 3: Transferable skills training

Pillar 3 is dedicated to the development of transferable skills that are essential for Early Stage Researchers (ESRs) to thrive in both academic and non-academic environments. This pillar recognizes that, in addition to specialized scientific knowledge, researchers must possess a diverse set of skills that enable them to communicate, collaborate, and innovate effectively. The transferable skills training program is tailored to the unique needs and career paths of the ESRs and is delivered through joint industry-academia courses.

**Table 4.** Selected courses (E existing, N - new module) continued.

#	Courses (E existing, N - new module)	WP	Tutorial content	Lead	ECTS	Month
13	Digital Finance Regulation (E)	4	Overview of the regulatory field in digital finance. Outlook to pending changes in EU regulations. Directions and focus points. Best practices for compliance and monitoring.	ECB	3	M12
14	History and Prospects of Digital Finance (N)	4	Past developments in digital finance (including digital assets, algorithmic trading, AI) and trends for the next decade. Reflection on decentralisation. Reflection on AI.	UNA	3	M18
15	Blockchains in Digital Finance (E, N)	4	Technical, financial and legislative principles of blockchain technology and its (potential) applications in digital finance. Impact of decentralised finance.	HUB	4	M24
16	Digital EIT Summer School (E, N)	5	Disrupting Finance with Digital Technologies. Reflection on the impact of FinTech on society. Overview of latest advances. Case studies. Learning to write a business plan.	EIT	4	M18
17	Green Digital Finance (E, N)	5	Instill awareness of energy consumption and ecological footprint of digital finance. Techniques for energy-efficient algorithm training and deployment of digital finance services. Trade-offs between performance and environmental impact.	KUT	3	M24
18	Multi-Criteria Decision Making in Sustainable Finance (E, N)	5	Principles of multi-criteria decision making. Various techniques and concepts (e.g., fuzzy set theory, analytical hierarchy process, preference modelling) to incorporate multiple objectives, in line with ESG principles.	FRA	3	M30

*Notes*: This table continues the listing of selected courses, both existing (E) and new (N), in the program. It includes details on the course name, work package (WP), tutorial content, lead organization, European Credit Transfer and Accumulation System (ECTS) credits awarded, and the scheduled month. The courses cover various aspects of digital finance, including regulation, history and prospects, blockchain technology, green digital finance, and multi-criteria decision-making in sustainable finance.

***5.3.1 Tailored program.*** The transferable skills development program is designed to be flexible and responsive to the individual needs of the ESRs. It includes a combination of workshops, seminars, online courses, and hands-on training sessions. The program is delivered by experienced professionals from both academia and industry, ensuring a well-rounded perspective.

***5.3.2 Types of transferable skills.*** The training covers a wide range of transferable skills, including but not limited to:

- **Communication skills:** Effective writing, speaking, and visual communication for diverse audiences, including academics, industry professionals, policymakers, and the general public.
- **Project management:** Planning, executing, and managing research projects, including budgeting, scheduling, risk management, and stakeholder engagement.
- **Entrepreneurship and innovation:** Understanding the principles of entrepreneurship, intellectual property rights, commercialization of research, and innovation in a business context.
- **Ethics and integrity:** Awareness of ethical considerations in research, including responsible conduct, data privacy, and compliance with regulations.
- **Collaboration and teamwork:** Building and maintaining effective collaborations, working in multidisciplinary teams, and managing conflicts.
- **Career development:** Strategies for career planning, job search, networking, and personal branding.

***5.3.3 Alignment with the 2018 Eurodoc report.*** The transferable skills training program is aligned with the recommendations and guidelines outlined in the 2018 Eurodoc report for early-stage researchers ^9^. This alignment ensures that the training is consistent with recognized best practices and addresses the key competencies that are valued by employers and stakeholders in the research community.

***5.3.4 Integration with research training.*** The transferable skills training is integrated with the scientific training provided in Pillars 1 and 2. This integration ensures that ESRs are not only equipped with advanced scientific knowledge but also possess the complementary skills needed to translate their research into real-world applications, engage with diverse stakeholders, and pursue a wide range of career opportunities.

### 5.4 Pillar 4: Training through secondments

Pillar 4 emphasizes the importance of hands-on experience and real-world exposure in the training of Early Stage Researchers (ESRs). Through a structured program of secondments, ESRs will have the opportunity to spend time at world-leading partner research centers and in industry settings. This immersive experience is designed to broaden their perspectives, enhance their skills, and foster collaboration and innovation.

***5.4.1 Purpose of secondments.*** The secondments are intended to provide ESRs with a comprehensive understanding of how Digital Finance is implemented, regulated, and innovated across various sectors. By working closely with professionals in traditional financial intermediaries, innovative Fintechs, central banks, and leading research organizations, ESRs will gain insights into the practical challenges and opportunities in the field of Digital Finance.

***5.4.2 Structure of secondments.*** Each ESR will spend a total of four months at one of the world-leading partner research centers, including the European Central Bank (ECB), ARC, and Fraunhofer Institute. Additionally, they will spend 18 months in industry settings. The secondments are strategically scheduled to align with the ESRs’ individual research projects (IRPs) and career development plans.

***5.4.3 Partner research centers.*** The partner research centers play a crucial role in the secondments, offering unique opportunities for ESRs to engage in cutting-edge research, policy analysis, and technological innovation. Specific contributions include:

- **European Central Bank (ECB):** Exposure to banking supervision, statistics, macroprudential policies, financial stability, and international cooperation. Understanding how one of the leading central banks globally shapes and influences the financial landscape.
- **ARC:** Opportunities to work with world-class researchers on key future-relevant technologies, with a focus on commercialization and business applications.
- **Fraunhofer Institute:** Experience in applied research, innovation, and excellence in various domains related to Digital Finance.

***5.4.4 Industry experience.*** The industry secondments provide ESRs with a chance to work with both traditional financial intermediaries across Europe (DEL, INT, RAI, SWE) and innovative Fintechs (ROY, CAR). These experiences will allow ESRs to:

- Gain hands-on experience in implementing Digital Finance solutions.
- Understand the regulatory environment and compliance requirements.
- Collaborate with industry experts on real-world projects.
- Explore emerging trends, technologies, and business models in the financial sector.

***5.4.5 Benefits of secondments.*** The secondments offer numerous benefits, including:

- **Skill development:** Enhancement of technical, analytical, and problem-solving skills through practical experience.
- **Networking:** Building professional relationships with leading researchers, industry experts, and policymakers.
- **Interdisciplinary collaboration:** Working across disciplines to foster innovation and creativity.
- **Career advancement:** Exposure to diverse career paths and opportunities in academia, industry, and government.

### 5.5 International conferences and other training programs

***5.5.1 Network training events.*** Network training events are a vital component of the DIGITAL program, designed to foster collaboration, knowledge exchange, and professional development. These events are often combined with larger gatherings that include a diverse array of participants, such as industrial partners, civil society, policymakers, and scholars from outside the network. Specific features include:

- **Collaboration with international initiatives:** Integration with initiatives like COST Action CA19130, which involves 240 researchers from 39 countries, and ECMI, with 170 research organizations across Europe.
- **Community building:** Opportunities for doctoral candidates to build their research community, network with experts, and engage in interdisciplinary collaboration.
- **Knowledge dissemination:** Platforms for sharing research findings, methodologies, and innovations beyond DIGITAL, enhancing visibility and impact.

***5.5.2 Participation in international conferences.*** ESRs are encouraged to participate in renowned international conferences, workshops, and symposia. These include, but are not limited to:

- NeurIPS (Conference on Neural Information Processing Systems)
- AFA Annual Conference (American Finance Association)
- EFA Conference (European Finance Association)
- ECMI Conference (European Capital Markets Institute)
- International R Conference
- Apply(conf) (Applied Data Science Conference)
- Data Reliability Engineering Conference
- DeFi Conference (Decentralized Finance)

Participation in these events provides ESRs with opportunities to present their research, receive feedback from leading experts, explore cutting-edge developments, and establish professional connections.

***5.5.3 Lab training.*** DIGITAL offers extensive lab training through direct access to various specialized labs focused on data, analytics, blockchain, regulation, supervision, and more. The lab training includes:

- **Utilizing data:** Hands-on experience in handling, analyzing, and interpreting complex data sets.
- **Constructing models:** Training in building and optimizing Machine Learning (ML) and Reinforcement Learning (RL) models.
- **Validating results:** Techniques for validating and evaluating research findings, ensuring accuracy and reliability.
- **Innovation and exploration:** Access to state-of-the-art facilities like UTW and BFH’s Digital Finance labs, the ECB’s cloud-based digital virtual lab, Fraunhofer’s virtual reality lab, optimization lab, computer graphics and visualization lab, analytics lab, Deloitte’s analytics lab, WULABS for data analytics, ASE’s AI lab, Swedbank’s financial laboratory, and several more.

***5.5.4 Benefits and opportunities.*** The combination of network training events, international conferences, and lab training offers ESRs a multifaceted training experience. Benefits include:

- **Skill enhancement:** Development of technical, analytical, communication, and collaboration skills.
- **Networking:** Building relationships with researchers, industry professionals, policymakers, and other stakeholders.
- **Research exposure:** Opportunities to present and publish research, contributing to the global scientific community.
- **Career advancement:** Exposure to diverse career paths, collaboration opportunities, and potential partnerships.

This comprehensive approach ensures that ESRs are well-equipped with the knowledge, skills, and connections needed to excel in their research endeavors and future careers.

### 5.6 External scientific lecturers

DIGITAL features prominent external scientific lecturers from world-class institutes who provide insights into the state-of-the-art in digital finance subdomains and excellent networking opportunities.

### 5.7 Online courses

***5.7.1 Accessibility and flexibility.*** All course materials within the DIGITAL program will be made available online, offering a flexible and accessible learning environment. This approach ensures that Early Stage Researchers (ESRs) and other participants can access the courses from various locations, accommodating different schedules and learning preferences. Key features include:

- **Digital platform:** Courses will be hosted on a dedicated platform (geant.org), providing a centralized location for all educational resources.
- **Remote participation:** The option to follow courses online, allowing for participation from different geographical locations and enabling a broader reach.
- **On-demand access:** Recorded lectures, tutorials, and supplementary materials will be available for on-demand access, facilitating self-paced learning.

***5.7.2 Content creation and sustainability.*** A significant emphasis will be placed on creating new online content that aligns with the research objectives and training needs of the program. This content will be designed with long-term sustainability and reusability in mind:

- **Customized content:** Development of tailored online modules, tutorials, and lectures that cover specific topics related to Finance, data science, AI, ML, blockchain, sustainability, and more.
- **Collaborative development:** Engagement with faculty, industry experts, and researchers in content creation to ensure relevance, quality, and innovation.
- **Long-term reusability:** Creation of evergreen content that can be utilized by future cohorts, other educational programs, and the wider academic community.

***5.7.3 Benefits of online courses.*** The online course approach offers several benefits that enhance the learning experience and contribute to the overall success of the training program:

- **Inclusivity:** Enables participation from a diverse audience, including international students, working professionals, and those with mobility constraints.
- **Adaptability:** Allows for customization of learning paths, accommodating different learning styles and individual needs.
- **Scalability:** Facilitates the expansion of the program to reach a larger audience without significant additional resources.
- **Sustainability:** Contributes to the long-term impact of the program by creating a repository of valuable educational content.

### 5.8 Transferable skills courses

Our consortium recognizes the importance of equipping researchers with a diverse set of skills that extend beyond their specific research domains. To this end, we offer the following transferable skills courses, designed to foster professional development and prepare researchers for various career paths:

1. **Gender and diversity (M3, 2 ECTS):** This course focuses on the integration of gender and diversity dimensions in research. It aims to promote awareness and understanding of the importance of inclusivity in scientific inquiry. The course is led by the European Central Bank (ECB) and covers topics such as gender equality, cultural diversity, and ethical considerations in research.
2. **Research and project management (M12, 4 ECTS):** Targeting the essential skills required for effective research and project management, this course covers areas such as Project Management (ROY, DEL), Higher Education (HE) framework and research project management (HUB), Research Ethics and Sustainable Research Management (BFH), and Environmental Aspects (UNA). It provides comprehensive training in planning, executing, and evaluating research projects, with an emphasis on ethical considerations and sustainability.
3. **Research skills (M18, 4 ECTS):** This course is designed to enhance core research competencies, including Scientific Writing (BFH), Scientific Communication (RAI), Open Science Principles (UNA), and Citizen Science (WWU). It aims to improve researchers’ ability to communicate their findings effectively, adhere to open science practices, and engage with the broader community through citizen science initiatives.
4. **Entrepreneurship (M24, 4 ECTS):** Focusing on the entrepreneurial aspects of research and innovation, this course covers Intellectual Property Rights and Patenting (ECB, DEL), Entrepreneurship Training (EIT), Entrepreneurial Finance (BFH), and Start-ups and Industry Transfer (EIT, DEL). It provides insights into the commercialization of research, intellectual property protection, financial management, and collaboration with industry.
5. **Labor market skills (M36, 2 ECTS):** Recognizing the importance of preparing researchers for the labor market, this course offers training in Job Applications (UTW) and communication skills (UTW, DEL). It aims to enhance researchers’ ability to present themselves effectively in job applications and interviews, and to communicate confidently in professional settings.

These courses collectively provide a well-rounded education in transferable skills, ensuring that researchers are not only proficient in their specific research areas but also equipped with the competencies needed to thrive in various professional environments. The collaboration with renowned institutions and industry partners further enriches the learning experience, offering unique insights and practical knowledge that align with real-world demands.

## 6 Conclusion

The need for specialized training in the multifaceted field of Digital Finance has never been more pressing. This manuscript has outlined a comprehensive and innovative doctoral training program, DIGITAL, designed to meet the unique challenges and opportunities of the modern financial landscape. Through a structured approach encompassing four main pillars, the program offers a blend of scientific training, advanced research skills, transferable soft skills, and real-world industry experience. The inclusion of international conferences, online courses, and a focus on key areas such as AI, ML, Blockchain, and sustainability ensures a holistic and forward-thinking approach. By aligning with the strategic priorities of the European Union and integrating with leading institutions, DIGITAL promises to foster innovation, enhance competitiveness, and contribute to the digital and green transition of Europe. The program’s emphasis on collaboration, accessibility, and long-term sustainability positions it as a vital step towards shaping the future of Digital Finance education and research. The consortium’s offerings in transferable skills courses further enrich the curriculum, addressing essential aspects such as gender diversity, research ethics, entrepreneurship, and labor market skills. In summary, DIGITAL represents a significant advancement in the field, setting a new standard for interdisciplinary education and paving the way for the next generation of financial experts and innovators.

## Ethics and consent

Ethical approval and consent were not required.

## Data Availability

No data are associated with this article.
