# Supplementary material for: Towards a new PhD Curriculum for Digital Finance
Source: Open Res Eur. 2024 Jan 8;4:7. [Version 1] doi: 10.12688/openreseurope.16513.1 (PMC10831224; doi:10.12688/openreseurope.16513.1)
Supplement: Peer review history

# Reviewer report: Reviewer response for version 1

**DOI:** 10.21956/openreseurope.17827.r37477

**Published:** 2024-01-31

**Authors:** Roman Matkovskyy

**Affiliations:** 1 Department of Finance and Accounting, Rennes School of Business, Rennes, France

The submitted document is an open letter that discusses a new PhD curriculum for Digital Finance, offering an interdisciplinary approach to doctoral training and aiming to equip future researchers with the necessary skills and knowledge in the field of Digital Finance. The text outlines some main challenges in the field of Digital Finance and the need for the proposed PhD curriculum. It references several aspects of Digital Finance education and training and provides factual statements and supports many of these with citations. Most subject-specific terms and concepts are explained in the submitted text, that makes it understandable. 

 Overall, the curriculum integrates various disciplines, providing a well-rounded programme in Digital Finance, accounting for AI, blockchain technology, and sustainability/ethics, offering quite a balanced mix of theoretical knowledge, practical experience, and soft skills. Being a part of the COST Action it emphases international collaboration. The proposed curriculum aligns with the European Digital Finance Package and integrates well with leading institutions from the Network. 

 As the programme evolves, the following aspects could potentially be taken into consideration for its future development: (i) potentially, the introduction of a continuous improvement system and a 'closing the loop' mechanism, which could be useful for the further evolution of the programme; (ii) the inclusion of Programme Learning Objectives and their mapping, which can make it easier to visualize the 'building blocks' of the programme; (iii) in the future, some elective modules could potentially be introduced based on feedback.

Where applicable, are recommendations and next steps explained clearly for others to follow? (Please consider whether others in the research community would be able to implement guidelines or recommendations and/or constructively engage in the debate)

Yes

Does the article adequately reference differing views and opinions?

Yes

Are all factual statements correct, and are statements and arguments made adequately supported by citations?

Yes

Is the rationale for the Open Letter provided in sufficient detail? (Please consider whether existing challenges in the field are outlined clearly and whether the purpose of the letter is explained)

Yes

Is the Open Letter written in accessible language? (Please consider whether all subject-specific terms, concepts and abbreviations are explained)

Yes

Reviewer Expertise:

Traditional vs contemporary finance, AI and blockchain,  FinTech.

I confirm that I have read this submission and believe that I have an appropriate level of expertise to confirm that it is of an acceptable scientific standard.

# Reviewer report: Reviewer response for version 1

**DOI:** 10.21956/openreseurope.17827.r37475

**Published:** 2024-01-31

**Authors:** Albulena Shala

**Affiliations:** 1 Faculty of Economics, University of Prishtina "Hasan Prishtina", Pristina, Kosovo (Serbia and Montenegro)

The text presents a thorough and cutting-edge PhD training program in digital finance that answers to the various needs of the European finance sector, initiatives that support the development of communities, and cooperation with other parties. In order to guarantee accessibility and the long-term viability of the training materials, online courses are also available. Through specialized training and cross-sector engagement, training in digital finance seeks to give researchers the knowledge and abilities necessary to propel innovation and shape the direction of digital finance.

 In my answer, "partly," you have to write the abbreviations when you mention them for the first time. For example, ESRs. Please see the others as well.

Where applicable, are recommendations and next steps explained clearly for others to follow? (Please consider whether others in the research community would be able to implement guidelines or recommendations and/or constructively engage in the debate)

Yes

Does the article adequately reference differing views and opinions?

Yes

Are all factual statements correct, and are statements and arguments made adequately supported by citations?

Yes

Is the rationale for the Open Letter provided in sufficient detail? (Please consider whether existing challenges in the field are outlined clearly and whether the purpose of the letter is explained)

Yes

Is the Open Letter written in accessible language? (Please consider whether all subject-specific terms, concepts and abbreviations are explained)

Partly

Reviewer Expertise:

Fintech, Banking, Digital Finance

I confirm that I have read this submission and believe that I have an appropriate level of expertise to confirm that it is of an acceptable scientific standard.
